# Dissecting the molecular basis of variability for flowering time in *Camelina sativa*

**DOI:** 10.1111/pbi.70049

**Published:** 2025-03-20

**Authors:** Liyong Zhang, Venkatesh Bollina, Peng Gao, Isobel A. P. Parkin

**Affiliations:** ^1^ Agriculture and Agri‐Food Canada Saskatoon Saskatchewan Canada

**Keywords:** Camelina, flowering time, pan‐transcriptome, genetic variation, association analyses

## Abstract

*Camelina sativa* is an important polyploid oilseed crop with multiple favourable agronomic traits. Capturing the leaf transcriptome of 48 accessions of *C. sativa* suggests allelic variation for gene expression levels and notably sub‐genome dominance, both of which could provide opportunities for crop improvement. Flowering time (FT) is a crucial factor affecting the overall yield of crops. However, our understanding of the molecular mechanisms underlying FT regulation in *C. sativa* are still limited, partly due to its complex allohexaploid genome. In this study, weighted gene co‐expression network analysis (WGCNA), expression quantitative trait loci (eQTL) analysis and transcriptome‐wide association study (TWAS) were employed to explore the FT diversity among 48 *C. sativa* accessions and dissect the underlying molecular basis. Our results revealed a FT‐related co‐expressed gene module highly enriched with *SOC1* and *SOC1*‐like genes and identified 10 significant FT‐associated single nucleotide polymorphisms (SNPs) defining three haplotype groups; thus providing a molecular basis for future genetic improvements in *C. sativa* breeding.

## Introduction

Like the model plant *Arabidopsis thaliana*, *Camelina sativa* is a member of the Brassicaceae family. Due to the sustainable and adaptable nature of the crop, over the past decade *C. sativa* has gained considerable attention as an important oilseed to support the food, feed and biofuel industries (Berti *et al*., 2016; Vollmann and Eynck, 2015). The genome sequence of *C. sativa* was published in 2014 (Kagale *et al*., 2014) and a transcriptome atlas detailing gene expression across a range of tissues has been developed (Kagale *et al*., 2016), providing a knowledge base for further development of the crop. Yet trait improvement relies on access to underlying genetic variation and a number of genotyping studies have shown limited variation within available *C. sativa* germplasm (Chaudhary *et al*., 2020; Gehringer *et al*., 2006; Luo *et al*., 2019; Singh *et al*., 2015). A recent assessment of genetic diversity in the largest collection of lines to date concluded that *C. sativa* has a modest degree of genome diversity, comparable to that of related *Brassica napus* (Li *et al*., 2021). This would concur with the evolutionary trajectory of the two crops, both being recent neopolyploids, probably formed from a limited number of hybridization events. Despite the modest level of genome diversity, Li *et al*. (2021) identified a wide range of phenotypic variation for a number of important agronomic traits. The phenotypic variation could stem from the complex control of many traits by a large number of small effect loci, which was suggested by the inherent difficulty in applying genome‐wide association analyses (GWAS) in *C. sativa* (Li *et al*., 2021).

At the level of gene expression, there is limited information available for variation among *C. sativa* accessions with data collected from only a handful of lines (Gomez‐Cano *et al*., 2020). Many lines of evidence implicate gene regulation as the source of variation for a large number of traits and the application of GWAS based on transcriptome data has identified useful loci controlling traits in species with recognized low‐genetic diversity, such as *B. napus* (Harper *et al*., 2012; Havlickova *et al*., 2018; Tang *et al*., 2021). In the current study, transcriptome data were collected for 48 publicly available accessions of *C. sativa* in order to assess the level of available variation and to provide an exploitable resource for researchers.

For plants, the transition from vegetative to reproductive phase, or flowering, is one of the most important developmental switches from the perspective of reproductive success. Regulation of flowering has been investigated over many decades in the model plant *Arabidopsis thaliana* and other flowering plants. Tremendous progress has been made with six different flowering pathways established; namely, autonomous, vernalization, photoperiod, aging, gibberellin (GA) and thermosensory pathways (Kim, 2020). In addition, there are complex interactions between the pathways, which determine reproductive success. For instance, both the autonomous and thermosensory pathways promote the floral transition through the main floral repressor, FLOWERING LOCUS C (FLC) (Amasino and Michaels, 2010; Capovilla *et al*., 2015; Cheng *et al*., 2017; Sheldon *et al*., 2000; Simpson, 2004). Increasing evidence has shown these floral pathways converge on several common genes, well‐known as floral integrators, including *FLOWERING LOCUS C* (*FLC*), *CONSTANS* (*CO*), *SUPPRESSOR OF OVER EXPRESSION OF CONSTANS* (*SOC1*), *FLOWERING LOCUS T*, *SHORT VEGETATIVE PHASE* (*SVP*) and *LEAFY* (*LFY*) to fine‐tune flowering time (Araki, 2001; Komeda, 2004; Lee *et al*., 2007; Moon *et al*., 2005; Srikanth and Schmid, 2011; Turck *et al*., 2008). In addition, epigenetic pathways and small non‐coding RNAs have also been shown to play important roles in flowering time regulation by altering expression of *FLC* (Teotia and Tang, 2015; Wang and Kohler, 2017). Thus, flowering plants have developed an elaborate network of genetic pathways responding to both exogenous environmental stimuli (e.g. temperature and photoperiod) and endogenous cues (e.g. hormone, sugars) to regulate floral transition.

However, for the allohexaploid *C. sativa*, a close relative of *A. thaliana*, there have been limited studies of how flowering time is regulated. Given that the floral integrator genes (e.g. *FLOWERING LOCUS T* and *SOC1*) are highly conserved among most of the flowering plants and the high‐collinearity between *C. sativa* and *A. thaliana* genomes (Kagale *et al*., 2014), researchers have attempted to understand the molecular basis underlying flowering time in *C. sativa* through isolating homologues of key flowering time genes. Leveraging available genetic and genomic tools, a number of studies have identified the orthologues of the key floral repressor gene *FLOWERING LOCUS C* (*FLC*) in *C. sativa* as playing an important role in the flowering habits of *C. sativa* (Anderson *et al*., 2018; Chao *et al*., 2019; Chaudhary *et al*., 2023). Luo Lily *et al*. (2021), reported 20 significant flowering time‐associated single nucleotide polymorphisms (SNPs) through a single genome‐wide association study (GWAS) (Luo Lily *et al*., 2021). As the natural diversity of *C. sativa* provides an important revenue for positive trait selection and large variation in flowering time has been documented among different *C. sativa* accessions (Berti *et al*., 2016; Chao *et al*., 2019; Luo Lily *et al*., 2021); in this study, we exploited the developed transcriptome resource from the 48 different genotypes by integrating WGCNA, eQTL and TWAS analysis to dissect the molecular basis underlying flowering time (FT) regulation among these lines. Our results provide insights into the regulatory mechanisms underlying *C. sativa* FT trait and present 10 FT‐associated SNPs, which could provide a basis for modifying FT during future *C. sativa* breeding.

## Results

### Gene expression analyses among *C. sativa* accessions

**Figure 1:**
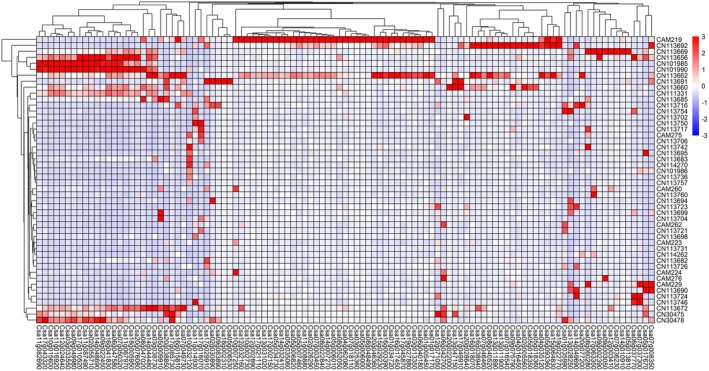
Dominantly expressed genes across the 48 *C. sativa* accessions. One hundred and seven highly variable genes (x‐axis) were identified based on TAU index (>0.95) and filtering for low expression, genes remaining have a mean normalized count >50 across all replicates in at least one accession. The genes were Z‐score normalized and then visualized using hierarchical clustering combined with a gene expression heatmap. The colour scheme, from red through white to blue, indicates the level of normalized expression, from high to low across 48 *C. sativa* accessions (y‐axis).

To facilitate the investigation of the role of gene regulation in controlling expression of agronomic traits RNA sequencing was conducted with young leaf tissue from 48 genotypes. Overall, approximately 926 million clean reads were obtained, with an average number of 18.9 million reads per genotype. All clean reads were aligned against the *C. sativa* reference genome, with mapping rates ranging from 72.5% to 82.9%. After quantifying the gene expression levels, of the total 94 495 *C. sativa* gene models, 37 268 were detected across all 48 genotypes and 72 713 genes were expressed in at least at one genotype, while the numbers of expressed genes varied between different genotypes ranging from 45 642 (CN113724) to 55 582 (CN113692) (Figure S1A). The gene expression data provides an indication of the level of variability in the population. Treating the data as a pan‐transcriptome dataset identified a subset of 184 genes that were significantly overrepresented in 10 or fewer of the lines (TAU > 0.9) (Figure 1; Figure S2). GO annotation enrichment of this gene set showed a significant overrepresentation of genes involved in stress responses, in particular in plant defence (Figure S3). Interestingly some lines show expression of a high proportion of these defence genes.

**Figure 2:**
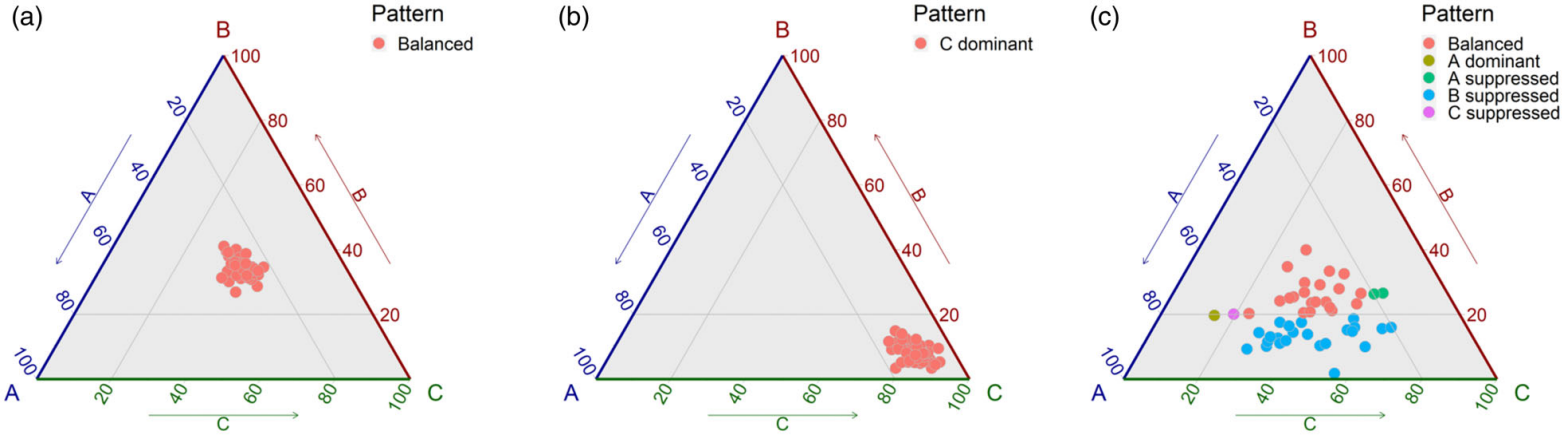
Patterns of genome dominance in 48 *C. sativa* accessions. The vertices of the triangle, A, B and C, represent the three orthologous gene copies in the sub‐genomes of *C. sativa*, respectively. Each filled circle represents the relative level of expression for each of the three genes and are coloured according to the shown legend. (a) Balanced gene expression for Csa11g001340, Csa10g001220 and Csa12g001280; (b) Sub‐genome 3 genome dominance for triad Csa11g101280, Csa18g037800 and Csa02g072860; and (c) Divergent genome dominance among accessions for triad Csa14g063510, Csa03g060290 and Csa17g093410; orthologues of At1g53160 or SPL4, a FT gene.

The polyploid nature of the *C. sativa* could provide another mechanism for creating expression diversity, since differential sub‐genome bias or genome dominance is a common trait in polyploids and has previously been reported for the *C. sativa* reference genome (Kagale *et al*., 2014). Considering the 12 440 syntenic triads, consisting of orthologous genes from the three sub‐genomes with some level of gene expression, 2101 showed balanced genome expression across the 48 accessions and 2460 showed the same pattern of genome dominance across the 48 lines (Figure 2a). The remaining triads showed differential patterns of genome dominance biased to one of the three sub‐genomes among the 48 accessions (Figure 2b). For those triads where the bias was not conserved among the accessions, 284 showed significantly divergent expression patterns with less than half of the accessions displaying the same pattern of dominance (Figure 2c). Annotation of these divergent genes identified a wide range of transcription factors, potentially impacting a number of traits including flowering time (Figure 2c).

### Generation of genome‐wide gene‐associated SNPs

**Figure 3:**
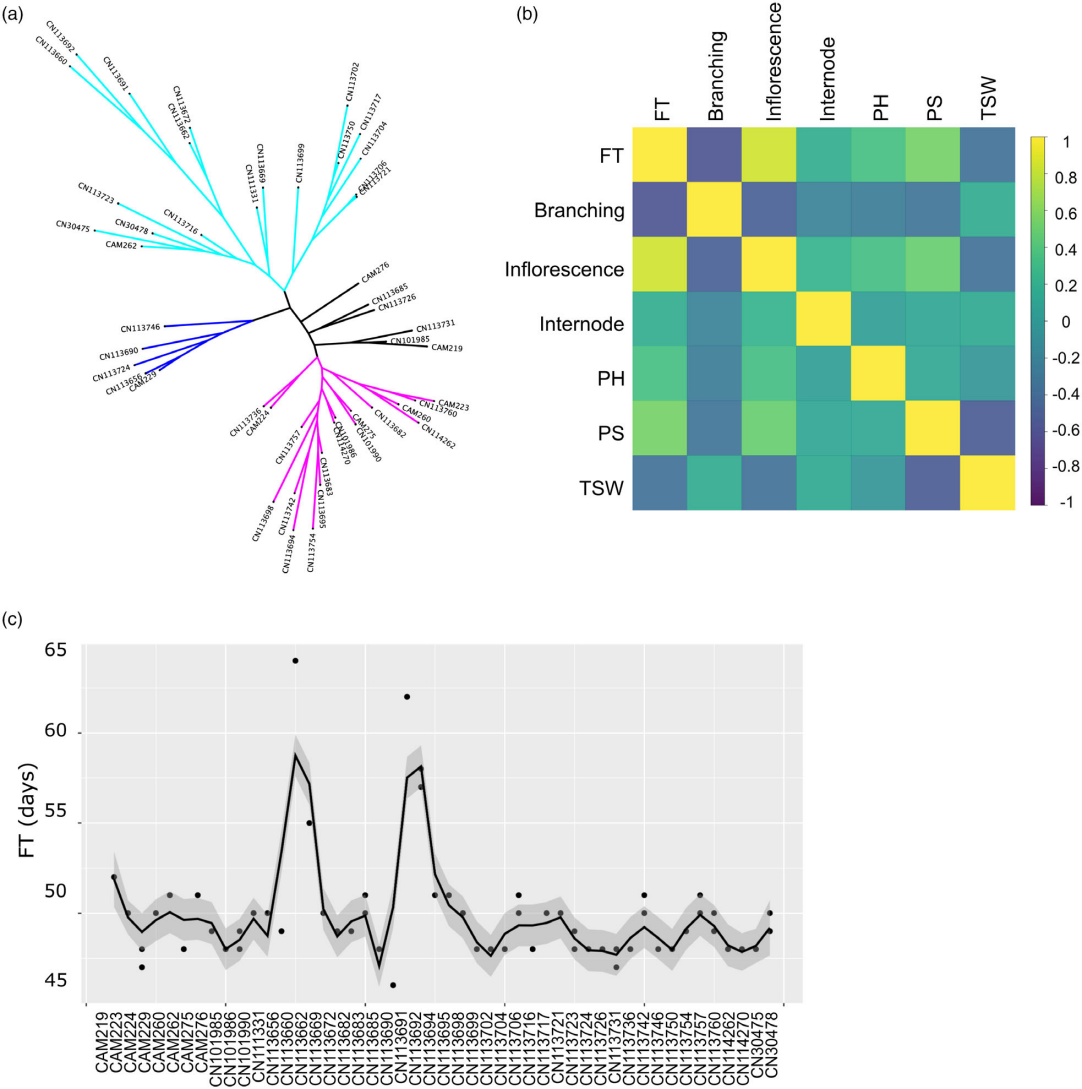
*Camelina sativa* population structure and FT variation. (a) Phylogenetic tree of all 48 experimental genotypes according to the final 65 082 SNPs. (b) Heatmap showing Pearson's correlation between different traits. Flowering time, plant height, pod shattering and 1000‐seed weight represented by the abbreviation FT, PH, PS and TSW, respectively. (c) Smoothed line plot of FT across all 48 genotypes in 2012.

To generate genome‐wide gene‐associated single nucleotide polymorphisms (SNPs), all clean reads of above RNA sequencing were aligned against the *C. sativa* reference genome to generate bam files, which were used for calling SNPs through genome analysis toolkit (GATK). In total, 1 151 672 SNPs were identified, which were further filtered to 65 082 SNPs. Among the 65 082 SNPs, the majority (56 469) were located within exons and 6865 SNPs were in 3′ or 5′ untranslated regions, while introns contained the remaining 1748 SNPs (Figure S1B). The small number of SNPs identified in introns were captured from genes with differentially spliced transcripts from those of the annotated reference genes. The final 65 082 SNPs were used to perform a phylogenetic analysis which resolved the 48 genotypes of the experimental population into 4 distinct groups (Figure 3a).

### Trait variation across *C. sativa* population

The 48 accessions were grown in the field for two consecutive seasons in 2012 and 2013, and data for a number of traits were collected, including flowering time (FT), inflorescence and internode length, plant height, 1000‐seed weight, pod shattering and branching (Data S1). The pairwise correlations between different traits were calculated using their best linear unbiased prediction values. As shown in Figure 3b, there was a significant positive correlation between FT and inflorescence length with a correlation coefficient of 0.84 (*P*‐value < 3.4 × 10^−14^), suggesting these two traits are likely mutually dependent. Meanwhile, we also noticed a significant negative correlation between FT and branching with a correlation coefficient of −0.57 (*P*‐value < 2.2 × 10^−5^) (Figure 3b). In the current study, we have focused on FT during *C. sativa* development. To check FT variation across the two seasons, their correlation was calculated as Pearson's correlation coefficient (*r* = 0.94 and *P*‐value < 2.2 × 10^−16^), suggesting a high consistency for FT between the two environments in 2012 and 2013. For clarity, only data from year 2012 was shown in Figure 3c, whereat FT varies largely between different genotypes of *C. sativa*, for example CN113690 only needs 46 days to reach 50% flowering on average, while CN113660 needs 8 more days (Figure 3c; see same trend of 2013 in Figure S1C).

### Identification of FT‐related highly co‐expressed gene modules

**Figure 4:**
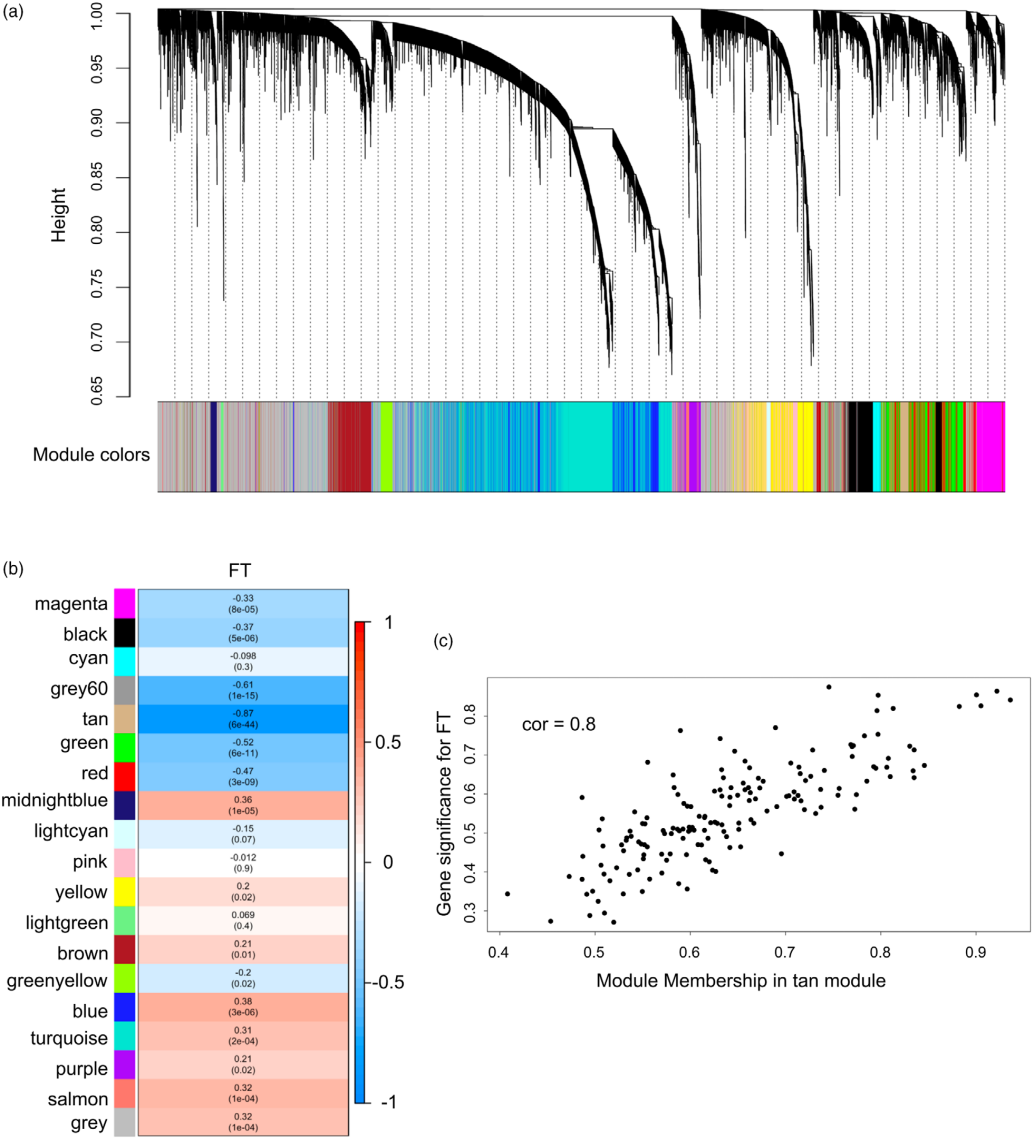
WGCNA identifies FT‐related co‐expressed gene module. (a) Hierarchical clustering dendrogram showing 19 distinct co‐expression modules based on 15 000 most variable genes across 48 genotypes. Different colours were assigned to each module. (b) Heatmap showing the correlation of FT with each co‐expression module. Each cell contains the corresponding Pearson's correlation coefficient and *P*‐value. (c) Scatterplot showing gene significance for FT and module membership in tan module.

Weighted gene co‐expression network analysis (WGCNA) provides a useful method to construct a co‐expression network, where each module of the network contains a group of genes with highly correlated expression patterns, which tend to be involved in similar biological processes (Langfelder and Horvath, 2008). To explore the potential co‐expressed gene module responsible for the above FT variation, we employed WGCNA to analyse the 15 000 genes with the highest expression variance across the experimental population. In the co‐expressed network, 15 000 genes were divided into 19 distinct modules (Figure 4a) with a size range from 46 to 4194 genes (Table 1). To quantify whether these modules associate with the FT trait, the Eigen genes (i.e. those genes representative of gene expression profiles in a module) of all 19 modules were used to calculate their Pearson's correlation coefficients with the FT trait (Figure 4b). As seen in Figure 4b, FT is highly negatively correlated with the tan module, which contains 164 *C. sativa* genes (Table 1). To further explore the genes in the tan module, the definitions from (Zhang and Horvath, 2005) were adapted, whereat the absolute value of the Pearson correlation between each gene and FT was defined as gene significance (GS), and module membership (MM) was defined by the individual gene's correlation with the Eigen gene of the tan module. By quantifying the GS and MM for each gene in the tan module and plotting their GS against MM, it was clear that there was a strong positive correlation between GS and MM, which indicated that genes related to FT were also the central genes of the tan module (Figure 4c).

**Table 1:** Co‐expressed gene modules and their gene numbers resulting from WGCNA

Module	Grey	Turquoise	Blue	Brown	Yellow	Green	Red	Black	Pink	Magenta
Number of genes	4194	3289	1133	1005	772	738	663	654	572	492
	Purple	Greenyellow	Tan	Salmon	Cyan	Midnightblue	Lightcyan	Grey60	Lightgreen	
	355	341	164	160	140	115	87	80	46	

**Figure 5:**
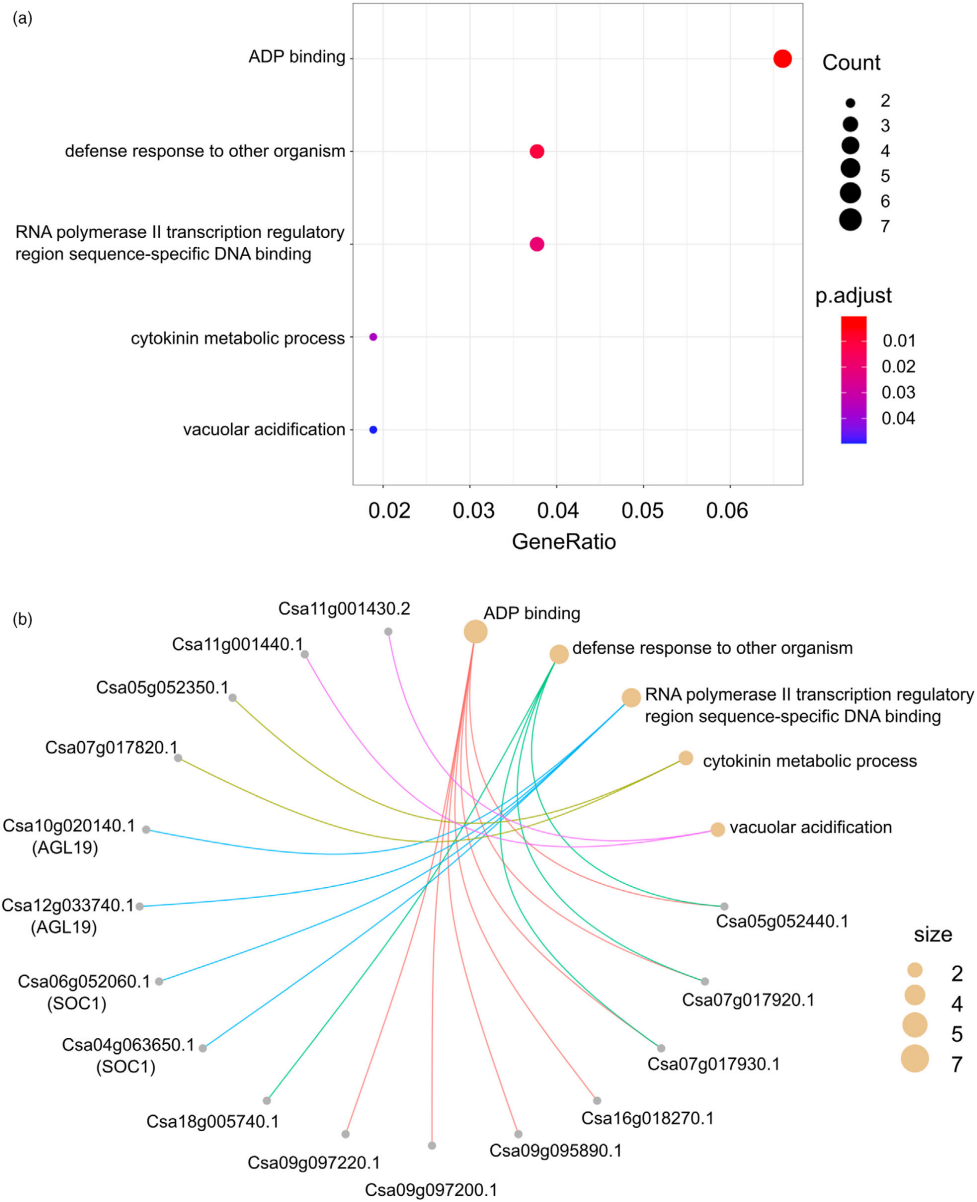
GO enrichment analysis of tan module. (a) Dot plot showing the 5 significantly enriched GO terms of tan module. Colour bar indicates the *P*‐values from Fisher's exact test, and dot size is the number of genes belonging to the given GO term. (b) Cnetplot listing details about which *C. sativa* genes associated with each GO term.

After generating the gene ontology (GO) database of *C. sativa* genes (see Methods for details), GO enrichment analysis through clusterProfiler was used to functionally characterize the tan module. Five GO terms were significantly enriched in the tan module (P‐values 0.05 after false discovery rate correction) (Figure 5a). Listing all genes assigned to the enriched GO categories showed that, the categories ‘defense response to other organism’ and ‘ADP binding’ shared multiple genes, while the remaining enriched GO terms did not overlap (Figure 5b). After further examination, it was noticed that all genes assigned to the categories ‘RNA polymerase II transcription regulatory region sequence‐specific DNA‐binding’ were members of the MADS transcription factor family, which are recognized for their role in floral organ development (Becker and Theissen, 2003). The MADS transcription factors enriched in the tan module are *AGL19* (AT4G22950) and *AGL20* (AT2G45660), both of which have been documented to participate in floral transition and promote flowering (Borner *et al*., 2000; Lee *et al*., 2000; Schonrock *et al*., 2006). Noticeably, *SOC1* (*AGL20*) is a well‐known key regulator of flowering timing and integrates different flowering pathways, and *AGL19* is classified as *SOC1*‐like gene according to its high similarity with *SOC1* (Becker and Theissen, 2003; Komeda, 2004). Due to the polyploid nature of *C. sativa*, the tan module contains more than one *SOC1*; Csa04g063650.1 and Csa06g052060.1 (Figure 5b; Data S2). More interestingly, the tan module also contains two *SOC1*‐like genes, *AGL19*s (Csa10g020140.1 and Csa12g033740.1), which suggests these *C. sativa SOC1* and *SOC1*‐like genes might work closely together in a similar manner as their Arabidopsis orthologues. Moreover, the tan module contained another MADS transcription factor *AGL103* (Csa07g033470.1) and additional characterized FT‐related genes, *SET DOMAIN PROTEIN 16* (*SDG16*; Csa10g015090.1), an AP2 transcription factor *SCHNARCHZAPFEN* (*SNZ*; Csa05g014000) and *SQUAMOSA PROMOTER BINDING PROTEIN‐LIKE 15* (*SPL15*; Csa09g070470.1). Thus, the network analysis identified a FT‐related co‐expressed gene module, which is highly enriched with MADS transcription factors, especially *SOC1* and *SOC1*‐like genes.

### eQTL and association analysis of tan module

As all 164 tan genes can be considered functionally FT‐relevant genes given their high correlation with the FT trait, an expression quantitative trait loci (eQTL) analysis was performed to identify putative eQTLs that have *cis* or *trans* regulatory effects on the expression of these FT‐related genes. During the eQTL analysis, all 65 082 SNPs were tested for their association with each of the 72 713 expression traits (i.e. gene expressed in least at one genotype). Accordingly, the Bonferroni adjusted *P*‐value threshold was calculated as 0.05/(65 082 × 72 713) = 1.06 × 10^−11^. At this threshold, in total 1769 SNPs (eQTLs) were identified as significantly associated with genes in the tan module.

**Figure 6:**
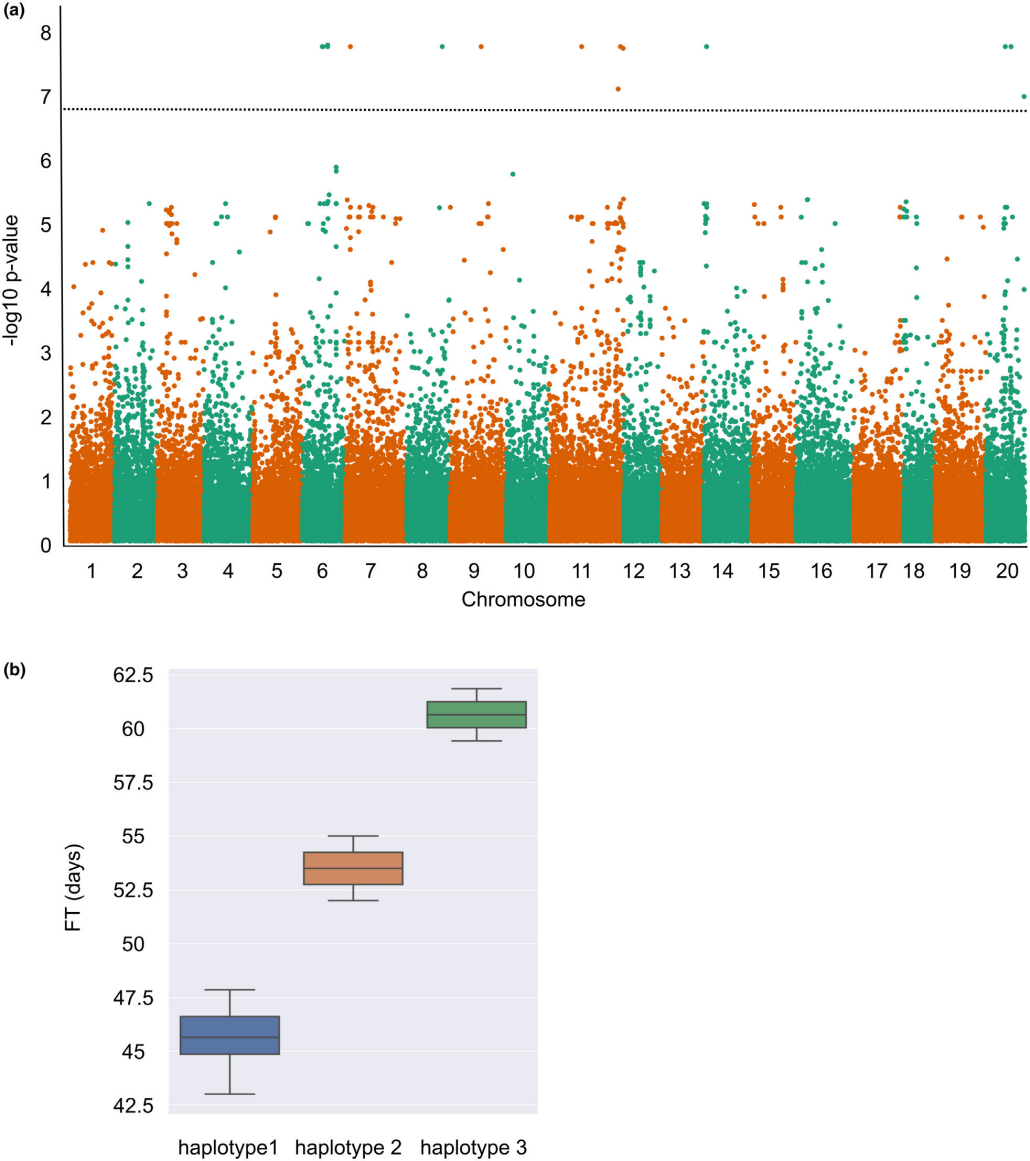
Association analysis identifies FT‐related SNPs. (a) Manhattan plot showing results from association analysis, The y‐axis represents –log(*P*‐values) of association between each SNP variant with FT trait, and the horizontal dotted line represents the threshold for significance. (b) Boxplot showing the FT values of the *C. sativa* accessions represented by each haplotype.

To further characterize the set of 1769 SNPs (eQTLs) above, we initially focused on SNPs that related with multiple well‐known FT genes included in the co‐expressed tan module, especially, *SOC1* and *SOC1*‐like genes. Seven SNPs were found to be associated with 2 *SOC1*s (Csa04g063650.1; Csa06g052060.1) and 1 *SOC1*‐like gene *AGL19* (Csa10g020140.1) (Data S3). Further, a transcriptome‐wide association study (TWAS) was used to mine the suggestive loci that were significantly associated with the FT trait. Employing a Linear Mixed Module in GEMMA, 10 out of the 1769 SNPs were confirmed to be significantly associated with FT (*P*‐value < 5 × 10^−7^; Figure 6a), which, however, did not include any of the seven SNPs that associated with multiple *SOC1* and *SOC1*‐like genes above. See Table 2 and Data S4 for a comprehensive view of these 10 significant FT‐associated SNPs, their related genes and corresponding annotations. Interestingly, when we examined these 10 SNPs, their distribution across the 48 experimental population show 3 distinct patterns, forming distributed haplotypes (Data S5). To further check their allelic effects on the FT trait, each individual haplotype was plotted against FT, which suggested that these 10 SNPs might help predict FT trait variation of *C. sativa* (Figure 6b). To conclude, by combining WGCNA, eQTL and TWAS analysis, we identified 10 important SNPs, which contribute to three different haplotypes, involved in FT variation in *C. sativa* plants.

**Table 2:** Significant SNPs associated with flowering time

SNP	Chr.	Pos.	Adjusted *P*‐value	Annotation	Associated gene	Encoded protein
snp_Chr6_18702229	6	18 702 229	1.42E‐08	missense_variant	Csa06g036830	Aquaporin NIP2.1
snp_Chr6_21176774	6	21 176 774	1.42E‐08	missense_variant	Csa06g042890	Jumonji/zinc‐finger‐class transcription factor protein
snp_Chr8_24560438	8	24 560 438	1.42E‐08	synonymous_variant	Csa08g057320	Encodes sorting nexin SNX2b
snp_Chr9_30922977	9	30 922 977	1.42E‐08	synonymous_variant	Csa09g081420	12‐oxophytodienoic acid reductase
snp_Chr11_18 992 361	11	18 992 361	1.42E‐08	synonymous_variant	Csa11g040110	Phytoene desaturase
snp_Chr11_47854078	11	47 854 078	1.42E‐08	synonymous_variant	Csa11g101310	Arabidopsis orthologue of human splicing factor SC35
snp_Chr14_2073315	14	2 073 315	1.42E‐08	synonymous_variant	Csa14g006410	Peptidase M50 family protein
snp_Chr20_8623095	20	8 623 095	1.42E‐08	synonymous_variant	Csa20g025800	Phosphate translocator family member
snp_Chr20_12463032	20	12 463 032	1.42E‐08	missense_variant	Csa20g036360	EMBRYO DEFECTIVE 1211; a MORN motif containing protein
snp_Chr20_12464248	20	12 464 248	1.42E‐08	missense_variant	Csa20g036360	EMBRYO DEFECTIVE 1211; a MORN motif containing protein

## Discussion

Multiple analyses have shown *C. sativa* to be limited in genetic diversity (Chaudhary *et al*., 2020; Gehringer *et al*., 2006; Singh *et al*., 2015); yet there has been documented evidence of phenotypic variability in available germplasm for a number of traits (Berti *et al*., 2016; Luo *et al*., 2019). Some of the latter variation could be explained by simple single gene control; however, it is probable that the multiple layers of genetic and epigenetic control deployed by most species are being exploited in *C. sativa* to allow further adaptation. In order to gain insights into the role of the transcriptome in providing opportunities for trait adaptation, gene expression was assessed in young leaf tissue across 48 accessions of *C. sativa*. These data showed distinct patterns of gene expression among the lines; although 37 268 core genes were expressed in all lines, between 8374 and 18 314 additional genes were variably expressed across the experimental population. Notably the most specifically expressed genes, found in ≤10 lines, were highly enriched for genes involved in plant defence; interestingly this observation aligns with that for plant pan‐genomes, where genes related to biotic and abiotic stress responses are commonly overrepresented among the annotated variable genes (Bayer *et al*., 2020). Although without full genome assemblies, we cannot rule out copy number variation (CNV) among the accessions, the transcriptome data suggests that CNV alone does not control trait variation in *C. sativa*. The polyploid genome structure of *C. sativa* also provides opportunity for trait adaptation; *C. sativa* has a highly undifferentiated hexaploid genome with most functional genes being represented by three orthologues (Kagale *et al*., 2014), providing opportunities for neofunctionalization of the duplicate gene copies. Among the transcriptome data 12 440 triads were identified and assessed for balanced or biased expression across the three sub‐genomes. Approximately one third (37%) of the triads showed conserved expression, either balanced or biased, across all of the lines, and as noted previously for *C. sativa* the third sub‐genome showed the highest level of genome dominance in all 48 accessions. The remaining triads showed evidence of differential bias among the accessions, providing opportunities for adaptation (Wang *et al*., 2022). Such variation has been shown to be of importance for crop improvement in polyploid wheat, with selection for particular yield traits resulting in genome imbalance associated with low‐expressing alleles of specific homoeologues (He *et al*., 2022). The prevalence of transcription factors among the triads showing the most variable patterns of bias among the accessions in *C. sativa* would suggest genome dominance could be a useful source of variation in this species. The cumulative transcriptome dataset provides a novel resource for studying trait variation in *C. sativa*, which was further queried to look for novel variation associated with FT in *C. sativa*.

### Floral genes in *C. sativa*

The floral integrator genes (e.g. *FLC* and *SOC1*) display very high conservation across flowering plants (Kim, 2020; Voogd *et al*., 2015). Further, the high levels of sequence homology and gene collinearity between *C. sativa* and Arabidopsis suggests *C. sativa* genes are expected to function in a similar manner as their Arabidopsis orthologous genes (Berti *et al*., 2016; Kagale *et al*., 2014). Study of a module highly correlated with FT from the co‐expression network analysis captured two of the three *C. sativa SOC1* orthologues, but not the third. This might suggest two *Csa.SOC1*s work in a concerted manner during FT regulation, while the third *Csa.SOC1* could have undergone neofunctionalization, as was suggested for *Csa.FLC* (Anderson *et al*., 2018). However, study of additional *Camelina* lines has identified a functional third copy of *Csa.FLC*, indicating that there may be variation yet to be discovered (Chaudhary *et al*., 2023). Interestingly, *SOC1*‐like genes (*AGL19*) also contributed two copies to the same co‐expressed gene module. In Arabidopsis, *AGL14*, *AGL19*, *SOC1* and *AGL42* belong to the *TM3*/*SOC1* clade based on phylogenetic analysis (Becker and Theissen, 2003). Not only do they share high‐sequence similarity, intensive interactions have also been shown between these *AGL*s, for example *SOC1* has been demonstrated to directly regulate the expression of *AGL42* during floral transition by balancing its expression level (Dorca‐Fornell *et al*., 2011). Further, *AGL42* activates the expression of all *AGL14/19/SOC1* genes during flower development, and all *AGL14/42/SOC1* genes show upregulated expression in the flowers of plants expressing 35S::AGL19, suggesting potential positive feedback loops between these *SOC1* and *SOC1*‐like genes, such that they reciprocally activate each other in order to regulate flowering time (Lee and Lee, 2010). However, there is likely to be a more complicated regulation landscape in *C. sativa* given that *C. sativa* possesses three copies of each of the *SOC1* and *SOC1*‐like genes (Kagale *et al*., 2014), adding another layer of complexity to the FT regulation network; how these *Csa.SOC1*s and *SOC1*‐like genes orchestrate to fine‐tune the FT of *C. sativa* will require further analyses.

### FT‐associated SNPs

The 10 FT‐associated SNPs could be resolved into three distributed haplotypes, one of which was dominant among the lines, while two lines carried haplotype 2 and an additional two lines haplotype 3; with number of days to flower increasing for those lines carrying the latter two haplotypes (Figure 6b). Since *FLC* is known to inhibit flowering by repressing *FLOWERING LOCUS T* and *SOC1*, and its role in conferring the winter habit in *C. sativa* has already been suggested (Chaudhary *et al*., 2023), the lines were assessed for expression at any of the three *FLC* loci. Interestingly, all lines showed a low level of expression from the *Csa.FLC.C08* locus, and no significant expression from the C13 and C20 loci, with the exception of the haplotype 3 lines, which showed significantly higher levels of *Csa.FLC.C20*, and the haplotype 2 lines, which showed significantly higher levels of *Csa.FLC.C13*. It appears that there is a natural variation for expression at the FLC loci, which may contribute to later flowering.

Based on the annotations of the identified 10 FT‐associated SNPs, there were two SNPs (snp_Chr20_12463032 and snp_Chr20_12464248) located to the same gene (Csa20g036360), suggesting a candidate haplotype block spanning at minimum the 1216 base pairs across this gene. Further BLASTP searches showed the orthologous gene of Csa20g036360 in Arabidopsis is AT5G22640, encoding EMBRYO DEFECTIVE 1211 (EMB 1211). Previous studies have shown that EMB 1211 is a key player involved in embryo development as well as chloroplast biogenesis (Liang *et al*., 2010; Tzafrir *et al*., 2004). Notably, constitutive expression of a maize *SOC1* (*ZmSOC1*) in soybean resulted in *EMB 1211* being highly upregulated, which suggested a possible feedback loop between SOC1 and EMB 1211 during plant development (Han *et al*., 2021). Interestingly, the correlation between the expression of Csa20g036360 and FT is very low (*r* = −0.12; *P*‐value = 0.17), which suggests its involvement in FT regulation is likely non‐linear in *C. sativa*.

Several other SNPs identified in this study merit further investigation to gain a more comprehensive understanding of their roles in the regulation of flowering time in *C. sativa*. One of the significant SNPs, snp_Chr6_21176774 was located within the region of gene Csa06g042890, whose orthologue in Arabidopsis encodes a Jumonji/zinc‐finger‐class transcription factor protein, namely JMJ19, which contains both Jumonji N (JmjN) and Jumonji C (JmjC) domains (Lu *et al*., 2008; Noh *et al*., 2004). Based on sequence similarities, there are a total of 9 putative JmjN/JmjC‐containing proteins in Arabidopsis, which are JMJ11, JMJ12, JMJ13, JMJ14, JMJ15, JMJ16, JMJ17, JMJ18 and JMJ19. These 9 JmjN/JmjC‐containing proteins are further divided into 2 sub‐groups, whereat JMJ11, JMJ12 and JMJ13 belong to the KDM4/JHDM3 group and the other six belong to the KDM5/JARID1 group (Lu *et al*., 2008). JMJ11 and JMJ12 are known as EARLY FLOWERING 6 (ELF6) and RELATIVE OF EARLY FLOWERING 6 (REF6) respectively, which have been reported to play divergent roles in flowering regulation in Arabidopsis (Noh *et al*., 2004); as their homologue, JMJ13 has also been shown to be a flowering repressor dependent on temperature and photoperiod (Zheng *et al*., 2019). Furthermore, JMJ14 acts through repression of floral integrators (i.e. *FLOWERING LOCUS T*, *AP1*, *SOC1* and *LFY*) to prevent early flowering during the vegetative phase (Jeong *et al*., 2009; Lu *et al*., 2010). As a large number of histone demethylases contain a conserved Jumonji C (JmjC) domain, these 9 proteins were predicted to be potential histone demethylases, and function in epigenetic regulation (Jeong *et al*., 2009; Lu *et al*., 2008, 2010; Zheng *et al*., 2019), which was affirmed by (Noh *et al*., 2004) where they showed JMJ12 functions as an FLC repressor through histone modifications of FLC chromatin. Therefore, it is possible that JMJ19 functions in a similar manner, as a histone demethylase during the regulation of flowering in *C. sativa*. Interestingly, snp_Chr11_47854078, locates to a region harbouring the orthologue of Arabidopsis *HUMAN SPLICING FACTOR SC35* (*AtSC35*). AtSC35 is a member of the serine/arginine‐rich (SR) proteins, which are important splicing factors (Barta *et al*., 2010). AtSC35 has been shown to play role in pre‐mRNA splicing along with another four SC35‐like (SCL) proteins. Further, AtSC35 and SCL proteins, in a redundant manner, control the transcription as well as the alternative splicing of *FLC* to regulate flowering time in Arabidopsis (Wang *et al*., 2023; Yan *et al*., 2017). Although stringent correction factors were applied, as with any association analyses further studies using additional genotypes, with diverse flowering times, will be needed to confirm the impact of any of the identified SNPs. It has been suggested that trait associated SNPs can increase the effectiveness of genomic selection, improving prediction accuracies for the target trait, thus the identified SNPs may prove useful in applied breeding strategies (Werner *et al*., 2018).

## Conclusion

FT is an important agricultural trait and understanding the mechanisms underlying its control could offer practical solutions in adapting FT to improve crop productivity in different environments. Owing to the identified allohexaploid genome, the genetic and molecular basis of FT are only beginning to be uncovered in *C. sativa*. In this study, WGCNA, eQTL and TWAS analysis were combined to dissect the FT trait in *C. sativa*; the results provide not only new insights into the genetic basis of *C. sativa* FT trait, but also offer new target genes for genetic improvement during future *C. sativa* breeding. Interestingly, the FT‐associated SNPs identified do not overlap those from a previous study (Luo Lily *et al*., 2021) but did show some correspondence to the expression of *FLC*. As our SNPs result from expression data, while (Luo Lily *et al*., 2021) carried out GWAS based on SNPs from genotyping‐by‐sequencing (GBS), this might explain the lack of correspondence. These results also indicate the complex genetic architecture underlying FT regulation in the allohexaploid plant *C. sativa* and emphasize the necessity of employing additional approaches to help us verify the core elements required for floral transition in *C. sativa*.

## Materials and methods

### Plant materials and phenotypic measurements

A *C. sativa* population comprising of 48 different accessions collected by Plant Gene Resources of Canada (PGRC, Saskatoon, SK, Canada) were used in this study. All accessions were planted in field trials at the experimental farm on Lowe Road, Saskatoon, during the growing season of 2012 and 2013. Three replicates were grown for each accession per season in double rowed plots. Flowering time (FT) was measured when 50% of plants had flowered in each plot. Inflorescence length, internode length and plant height were recorded as the average of measurements from three plants per plot. Mature pods and seeds were collected from three plants in each plot to measure 1000‐seed weight. The plants were left to mature in the field and pod shatter was rated subjectively on a scale of 1 to 10, with 1 being highly tolerant and 10 highly prone to shatter (no pod shells and only the pseudoseptum remaining).

### RNA extraction and sequencing

Forty‐eight different *C. sativa* accessions were cultivated under greenhouse conditions (16 h light/8 h dark, 22°). The leaf tissue from the young seedlings of *C. sativa* were collected at the stage when the fourth leaf was fully expanded to extract total RNA using the RNeasy plant mini kit (Qiagen, Toronto, ON, Canada) according to the manufacturer's protocol. The concentration and integrity of total RNA were examined by BioAnalyzer with RNA 6000 Nano Kit (Agilent Technologies, Inc., Santa Clara, CA, US). cDNA sequencing libraries were constructed according to the standard TruSeq RNA library preparation guide, and paired‐end sequencing was performed using the HiSeq 2000 platform (Illumina, Inc., San Diego, CA, US).

### Expression profiling and SNPs calling

Raw RNA‐seq data were trimmed using Trimmomatic (version 0.32) (Bolger *et al*., 2014) with the following settings (ILLUMINACLIP:TruSeq3‐PE.fa:2:30:10; SLIDINGWINDOW:4:15; LEADING:15; TRAILING:15; MINLEN:55) to remove adapter and low‐quality sequences. For expression profiling, clean reads were aligned to the *C. sativa* reference genome using STAR (version 2.7.10a) (Dobin *et al*., 2013) and gene expression levels were summarized by featureCounts (version 2.0.1) (Liao *et al*., 2014). Subsequent raw counts were filtered to remove lowly expressed genes (<1 across the samples) and then normalized through variance stabilizing transformation (VST) in DESeq2 (Love *et al*., 2014). The number of expressed genes across all 48 genotypes were calculated by assigning a gene as expressed only if the normalized counts were >3 in all three biological replicates.

For SNP calling, STAR's two‐pass mode was employed to map clean RNA reads to *C. sativa* reference genome. The binary alignment map (BAM) files were used to make unmapped BAM (uBAM) files through Picard's RevertSam function (version 2.27.4), filtered to remove PCR duplicates through Picard's MarkDuplicates function and further processed by the SplitNCigarReads function in GATK (version 4.2.6.1) (McKenna *et al*., 2010). The retained reads were used for variant calling through GATK's HaplotypeCaller, CombineGVCFs and GenotypeGVCFs functions with default parameters. Next, SNPs were collected using GATK's SelectVariants function, and filtered through the VariantFiltration function with setting ‘QD < 2.0 || MQ < 40.0 || FS > 60.0 || SOR > 3.0 || MQRankSum < −12.5 || ReadPosRankSum < −8.0’. Eventually, all 1 161 502 SNPs were further filtered through vcftools (version 0.1.16) (Danecek *et al*., 2011) with parameters ‘max‐missing 0.95, maf 0.05, hwe 0.000001’ and 65 082 SNPs were obtained for further analysis.

### Dominant expression/Genome bias

To identify the highly variable genes that are significantly overrepresented in select lines, TAU index was used for defining a gene's specific expression score. Pre‐filtering was performed to remove low‐expression genes with two criterions: (1) traditional mean normalized counts (>10); or (2) maximum normalized counts threshold (>50) across all accessions. After filtering low‐expression genes, TAU index of each gene from top expressed *n* lines (1 ≤ *n* ≤ 10) were calculated, respectively, and TAU > 0.9 was used as a threshold to define the dominantly expressed genes in a few lines. The highly variable genes identified based on TAU index were further visualized using hierarchical clustering combined with a gene expression heatmap performed by R pheatmap package (https://cran.r‐project.org/web/packages/pheatmap/index.html).

Normalized counts of 12 440 syntenic triads from all RNA‐seq samples were used to study the orthologous expression bias in all 48 *C. sativa* accessions based on the definition in a previous study (Ramírez‐González *et al*., 2018). With the thresholds defined as 20%, 80% and 100%, seven patterns for expression bias, including ‘Balanced’, ‘A dominant’, ‘B dominant’, ‘C dominant’, ‘A suppressed’, ‘B suppressed’, ‘C suppressed’, were identified based on the expression difference among the syntenic triads from the three sub‐genomes. All patterns were distinguished for the triads with at least one member holding an average expression of 10 normalized counts across all 48 *C. sativa* accessions. Ternary plots were used to visualize the relative expression contribution of each member of triads from three sub‐genomes and plotted by R ggtern package (Hamilton and Ferry, 2018).

### WGCNA and GO enrichment analysis

The VST normalized counts were further used to extract a subset (15 000) of the most highly variable genes across the experimental population through genefilter package in R. The selected subset of 15 000 most variable genes were used to construct a co‐expression network through the WGCNA package in R (Langfelder and Horvath, 2008), whereat the function blockwiseModules was used with parameters ‘power = 12, maxBlockSize = 15 000, networkType = ‘signed’, TOMType = ‘signed’, minModuleSize = 30, mergeCutHeight = 0.25’ to construct a signed network and generate co‐expressed gene modules. The *C. sativa* GO annotation database was created with its whole proteome using InterProScan 5 (Jones *et al*., 2014). Further, GO enrichment analysis was conducted for the module of interest using clusterProfiler in R (Wu *et al*., 2021).

### eQTL and association analysis

eQTL analysis was performed through Matrix eQTL in R (Shabalin, 2012). 65 082 SNPs and expression data from the selected subset of 15 000 most highly variable genes were used to perform the eQTL analysis. Significant eQTLs were defined as *cis* when the physical distance between SNP and its associated gene was <3 kb, and *trans* if the distance exceeded 3 kb. For transcriptome‐wide association study (TWAS), a mixed linear model through program GEMMA (version 0.98.5) (Zhou and Stephens, 2012) was carried out to detect the associations between 65 082 SNPs and FT's Best Linear Unbiased Predictors (BLUPs) values, which were generated using lme4 (version 1.1–34) in R. The first three principal components derived from all 65 082 SNPs were used as a covariate in the model to correct for population stratification.

## Conflict of interest

The authors have not declared a conflict of interest.

## Supporting information


**Data S1** Agronomic trait data from 2012 and 2013.

**Data S2** List of C. sativa genes in co‐expressed tan module.

**Data S3** SNPs associated with multiple SOC1 and SOC1‐L genes.

**Data S4** Annotations for FT‐associated SNPs.

**Data S5** Haplotype information for 10 FT‐related SNPs and associated FT data.

**Figure S1** Gene expression and FT across various genotypes and SNPs categories. (A) Gene expression variation across 48 *C. sativa* accessions. For each genotype, expressed gene was defined as its average expression of 3 replicates ≥5 vst normalized counts. (B) Pie plot shows the percentage of all 65 082 SNPs belonging to each category. (C) Smoothed line plot showing the FT variation across all 48 genotypes in year 2013.

**Figure S2** Dominantly expressed genes across the 48 *C. sativa* accessions. 184 highly variable genes (x‐axis) were identified, based on TAU index (>0.90) and filtering for low expression, genes remaining have a mean normalized count >10 across all replicates in at least one accession. The genes were Z‐score normalized and then visualized using hierarchical clustering combined with a gene expression heatmap. The colour scheme, from red through white to blue, indicates the level of normalized expression, from high to low across 48 *C. sativa* accessions (y‐axis).

**Figure S3** Enrichment for Biological Processes among Gene Ontology Annotation of most dominantly/specifically expressed genes.

## Data Availability

The raw RNA‐seq data have been deposited to the Gene Expression Omnibus (GEO) under the accession GSE253393. All other data are included in the article and/or supporting data.
