# Supplementary figures and images for: Dissecting the molecular basis of variability for flowering time in Camelina sativa
Source: Plant Biotechnol J. 2025 Mar 20;23(6):2290–302. doi: 10.1111/pbi.70049 (PMC12120899; doi:10.1111/pbi.70049)

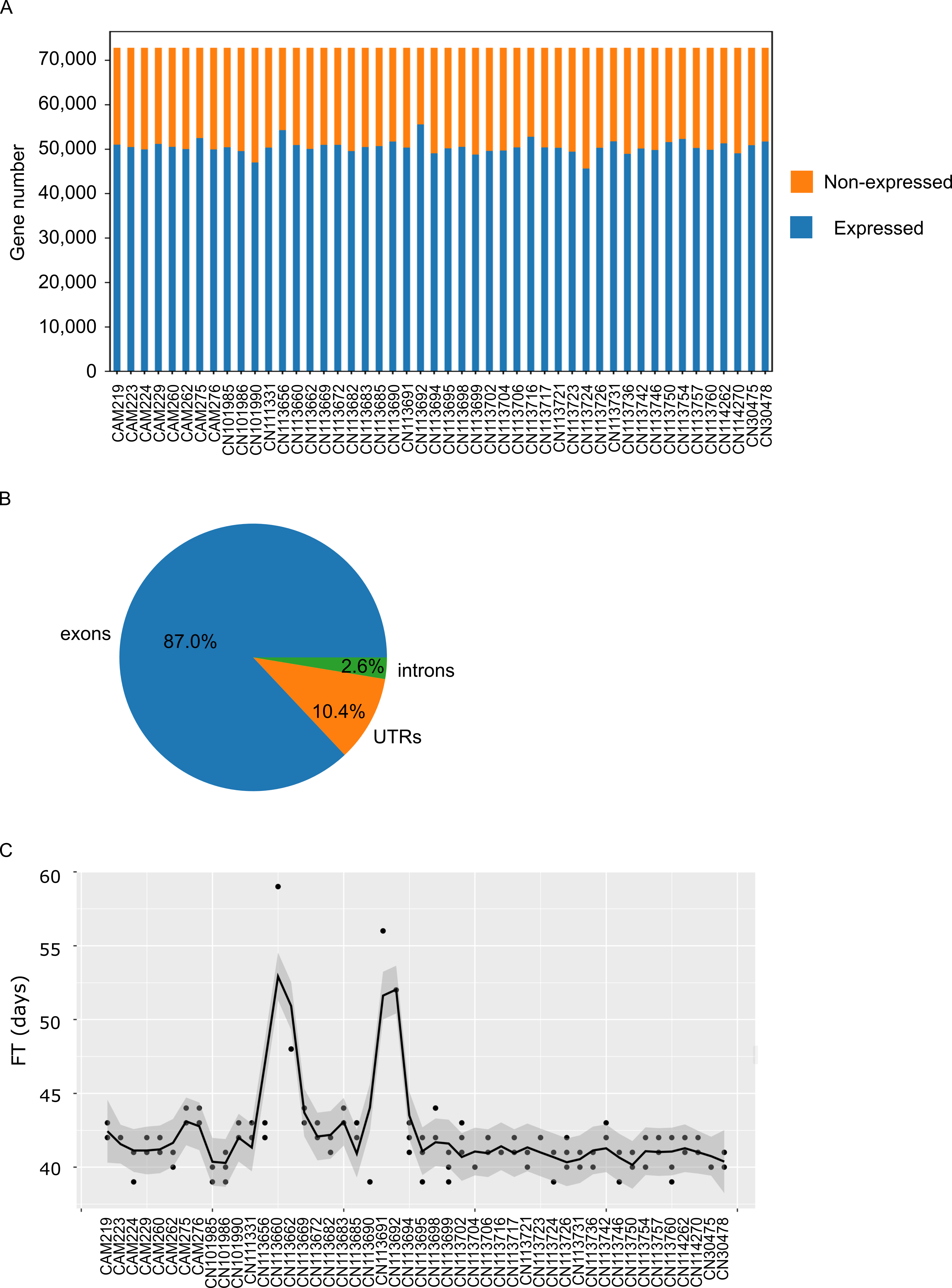

Supplement: Supplementary file 6 — Figure S1 Gene expression and FT across various genotypes and SNPs categories. (A) Gene expression variation across 48 C. sativa accessions. For each genotype, expressed gene was defined as its average expression of 3 replicates ≥5 vst normalized counts. (B) Pie plot shows the percentage of all 65 082 SNPs belonging to each category. (C) Smoothed line plot showing the FT variation across all 48 genotypes in year 2013. [file PBI-23-2290-s001.png]

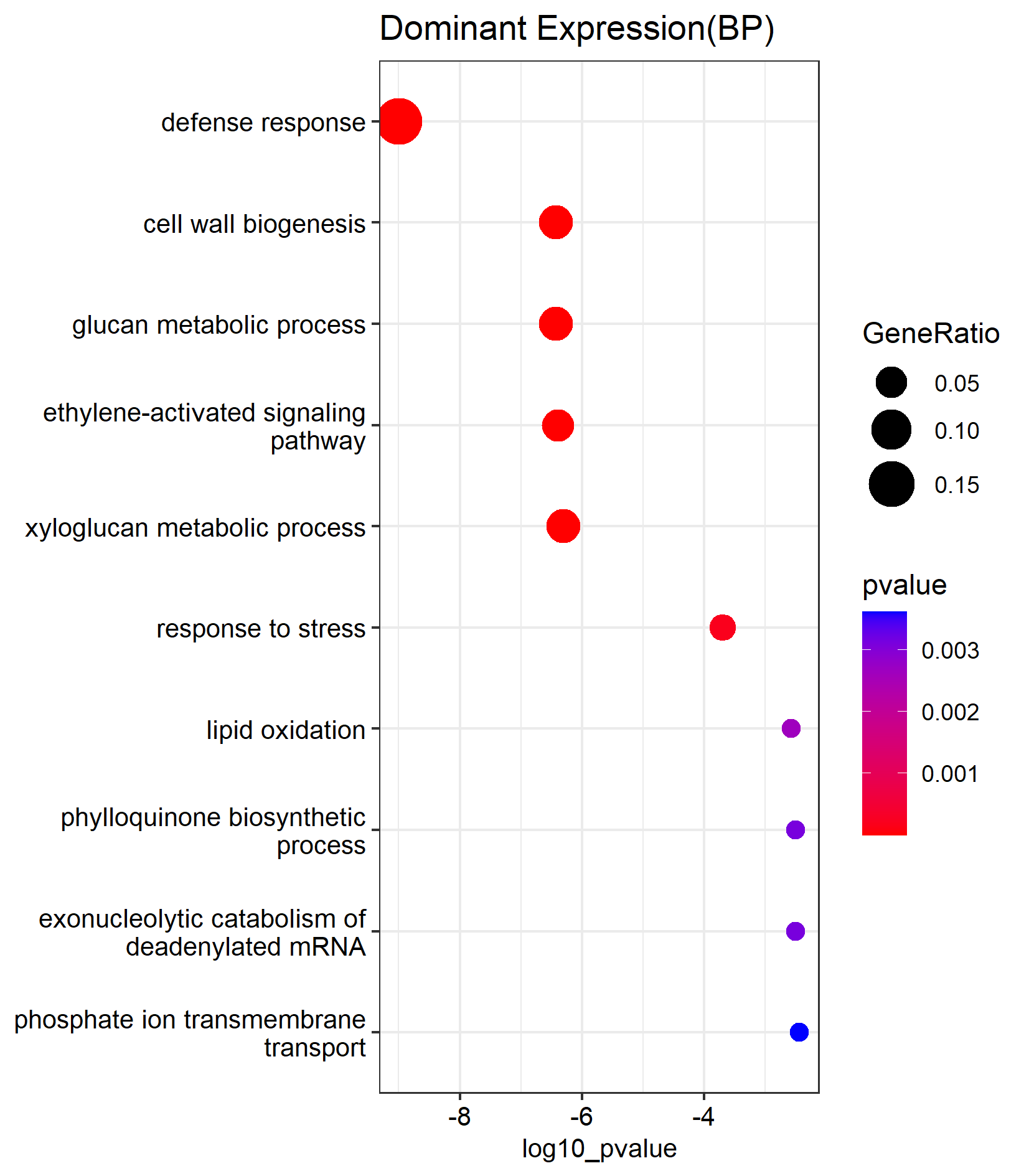

Supplement: Supplementary file 8 — Figure S3 Enrichment for Biological Processes among Gene Ontology Annotation of most dominantly/specifically expressed genes. [file PBI-23-2290-s006.png]
